# ERRATUM

**DOI:** 10.1111/1759-7714.14350

**Published:** 2022-03-01

The authors would like to draw the reader's attention to an error in the following article:

Zhang S, Zhang Y, Feng W, Shi Z, Shi H, Zhang Y. Prediction modeling using routine clinical parameters to stratify survival in malignant pleural mesothelioma patients complicated with malignant pleural effusion. Thorac Cancer. 2021; 12:3304–9. https://doi.org/10.1111/1759-7714.14202

On page 3307, the images of figures 2 and 3 should be interchanged. They should appear as:

**FIGURE 2:**
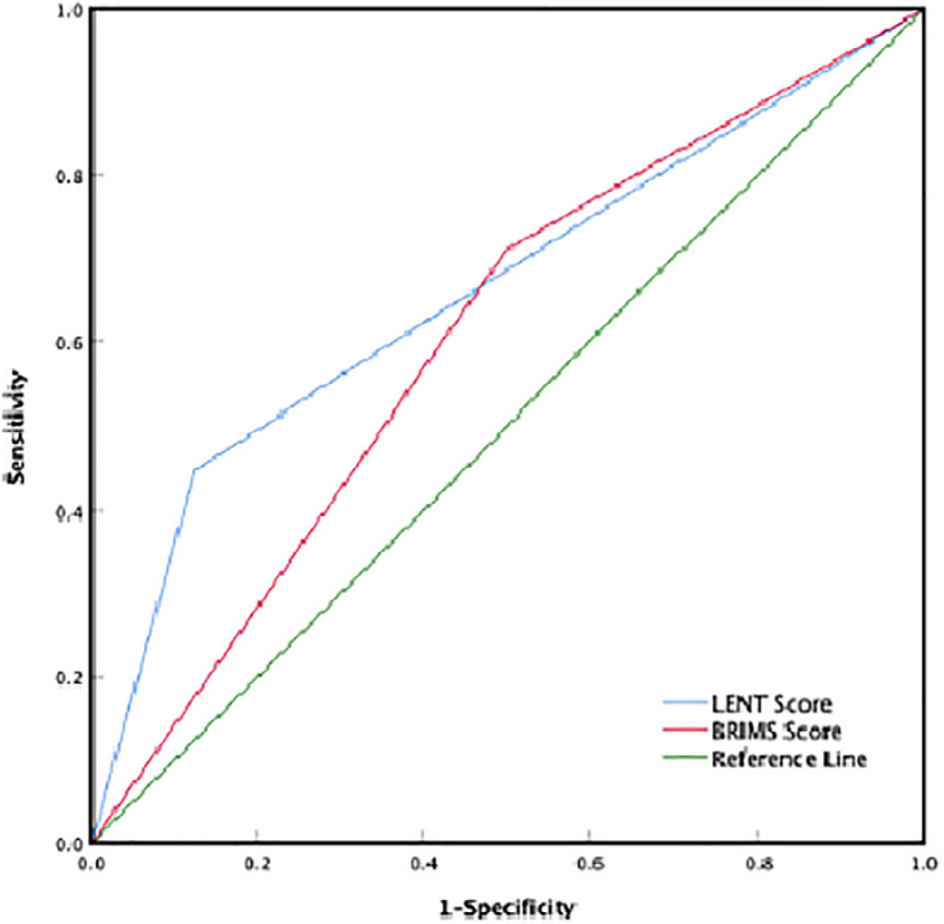
ROC curve of the LENT and BRIMS scores

**FIGURE 3:**
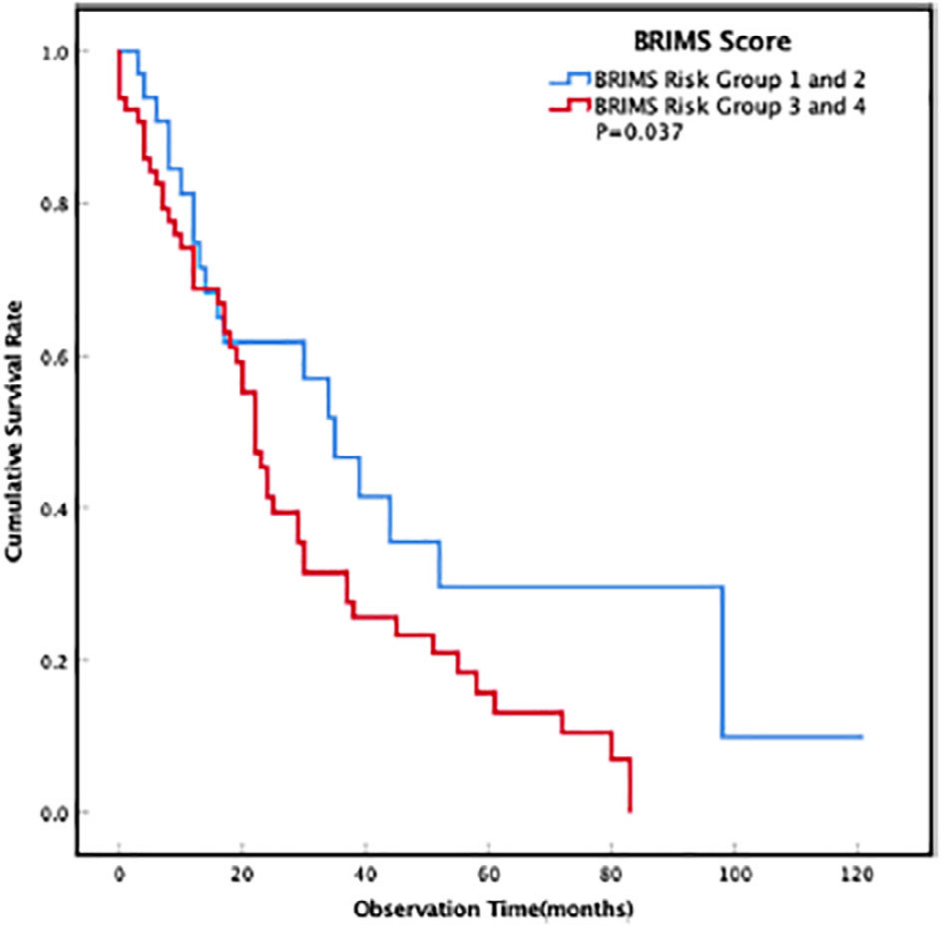
Comparison of overall survival among different risk groups of BRIMS using Kaplan–Meier curves survival analysis (*p* = 0.037)

The publisher apologizes for the error and any inconvenience it may have caused.

